# Assessment of the influence of Apolipoprotein E on the deposition of Arctic Aβ and seeded deposits in knock‐in and transgenic mouse models

**DOI:** 10.1002/alz70855_107455

**Published:** 2025-12-25

**Authors:** Conner D Angelle, Guilian Xu, Andrea Iturbe, Patricia Sacilotto, Susan Fromholt, Quan Vo, Karen McFarland, Stefan Prokop, David R. Borchelt, Paramita Chakrabarty

**Affiliations:** ^1^ University of Florida, Gainesville, FL, USA; ^2^ Mayo Clinic, Jacksonville, FL, USA; ^3^ University of Florida College of Medicine, Gainesville, FL, USA

## Abstract

**Background:**

Apolipoprotein E (apoE) facilitates the fibrillization and deposition of amyloid β (Aβ) in Alzheimer's Disease (AD). Supported by robust literature from various groups, in our current study we have investigated the relative amyloidogenic properties of the three major human APOE alleles. Knowledge from such studies could provide insight into the role of ApoE protein in the deposition, evolution and maturation of parenchymal Aβ.

**Method:**

We used hAbetaSAA (Jax Labs) mice crossed with human *APOE* targeted replacement mice to create hAbetaSAA mice homozygous for human *APOE E2, E3* and *E4*. Furthermore, APPsi mice (hAPPswe/indiana: Borchelt) expressing human *APOE E3* or *E4* were injected intracerebrally with AD brain lysates to examine the role of *APOE* genotype on Aβ seeding. Neuropathological examination was conducted via immunohistochemistry, immunofluorescence and immunoblotting.

**Result:**

hAbetaSAA mice expressing human *APOE E4* showed higher Aβ deposition relative to APOE *E3* and APOE *E2* by 5 months of age and progressing through 8 months and 13 months of age. In comparison, APP mice expressing humanized Aβ (Jax Labs) with human *APOE E4* did not develop deposits in their lifetime. hAbetaSAA mice with endogenous mouse *Apoe* had the highest Aβ plaque deposition, with an approximately 60‐fold increase in Aβ burden compared to SAA APP mice expressing *APOE E3*, especially in the earlier deposition phase. Removing mouse *Apoe* resulted in dose‐dependent reduction in Aβ deposition, consistent with previously published literature. Plaque morphology did not show any notable differences between *APOE* genotypes, and colocalization of apoE with Aβ were relatively equivalent across different *APOE* genotypes. Further, in the APPsi model seeded with AD‐derived seeds, we found that mice with APOE E4 or APOE E3 developed equal burdens of diffuse parenchymal Aβ, with no detectable changes in plaque morphology.

**Conclusion:**

We conclude that apoE variants broadly influence the rate of amyloid deposition, but do not modify the morphology of the resulting deposits or colocalization with Aβ. Notably, when seeding with AD patient brain lysate, there was no significant difference in Aβ burden between APPsi mice expressing *APOE E3* and *E4*. Our study adds insights into the role of apoE isoforms in determining Aβ‐related phenotype.

